# Pediatric systemic lupus erythematosus and human parvovirus B19 infection

**DOI:** 10.1186/1546-0096-9-S1-P267

**Published:** 2011-09-14

**Authors:** V Bélien-Pallet, S Sauvion, R Salomon, P Quartier, B Bader-Meunier

**Affiliations:** 1 Pediatric unit, Jean Verdier hospital, 93140 Bondy, France; 2 Pediatric Nephrology unit, Necker hospital, 75015 Paris, France; 3 Pediatric Immunology Rhumatology unit, 75015 Paris, France

## Aim

To describe presentation and course of paediatric systemic lupus erythematosus (pSLE), associated with Parvovirus B19(PB19) primo-infection.

## Methods

Retrospective monocentric study of patients with pSLE, defined according to ARA criteria, diagnosed before 16 years old, and associated with PB19 primo-infection, defined by the presence of PB19 IgM antibodies and PB19 DNA detected by PCR in blood and/or in tissue.

## Results

3 girls aged from 8 years to 15 years 8 months have been included. PB19 infection was present at diagnosis in all patients. Maternal history of autoimmune diseases was noted in a consanguineous family. Clinical manifestations at diagnosis were fever(3), arthritis/arthralgia (3), skin rash (1), grade IV glomerulonephritis (1), interstitial pneumonia (1), cerebral vasculitis (1), pericarditis/pleurisy (1), adenopathy (2), hepatomegaly/splenomegaly (1). Aregenerative anaemia requiring packed red blood cell transfusion was present in all patients. AAN (3), anti –DNA (3), anti-SSa (1), anti-SSb (1), anti-phospholipid (1), anti-platelet (2), rheumatoid factor (2), pANCA (1), anti-actin (1) antibodies were found. PB19 PCR was positive in blood (3), bone marrow (2) and kidney (1). Corticosteroids have been initiated for the 3 patients, in association with one or more immunosuppressive therapies. A sustained remission has been observed in 2 patients after a follow-up of 4 years and 1 year 8 months off-therapy respectively. A severe course with joint damages, recurrent pericarditis and gradeIV glomerulonephritis, associated with persistent infection by PB19, has been observed in the third case (family autoimmunity history).

## Conclusion

PB19 infection-associated-pSLE may evolve either to a persistent remission off-therapy or to a severe pSLE. It should be considered in patient with pSLE associated with a severe aregenerative anemia.

